# Cardiovascular Profiles for Phenotyping Cognitive Trajectories in the Irish Longitudinal Study on Ageing

**DOI:** 10.1093/geroni/igaa057.2294

**Published:** 2020-12-16

**Authors:** Celine De Looze, Wilby Williamson, Naiara Demnitz, Rose Anne Kenny

**Affiliations:** 1 Trinity College Dublin, Dublin, Ireland; 2 The Irish Longitudinal Study on Ageing (TILDA), dublin, Dublin, Ireland; 3 Global Brain Health Institute, Dublin, Dublin, Ireland; 4 The Irish Longitudinal Study on Ageing (TILDA), Dublin, Dublin, Ireland

## Abstract

Cardiovascular risk factors are increasingly recognized as modifiable risks for cognitive decline and dementia in later life. The Life’s Simple 7, an ideal CV health scoring system, has been recently put forward as a tool for the promotion of brain health. This study aims to evaluate the LS7 as risk prediction tool for cognitive decline trajectories (MMSE, immediate/delayed recall and verbal fluency) in 2,739 adults aged ≥50 years from TILDA. We investigate if indices of muscular strength (grip strength), mobility (Time Up and Go, walking speed) and physiological stress (e.g. orthostatic blood pressure and heart rate recovery) as add-ons to the LS7 improves prediction of cognitive trajectories; and, in a subcohort, we assess CV health score in association with multimodal brain measures. Identifying the factors that influence the onset and trajectory of cognitive decline in a multifactorial perspective is critical toward lowering dementia risks and developing adequate intervention and treatment.

